# Experimental Study on Mechanical Properties and Mix Design Optimization of Nano-SiO_2_-Double-Doped Fiber High-Strength Concrete

**DOI:** 10.3390/ma19071359

**Published:** 2026-03-29

**Authors:** Yanchang Zhu, Yanmei Zhang, Yingying Tao, Qikai Wang, Rui Zhang, Yongxiang Fang

**Affiliations:** College of Pipeline and Civil Engineering, China University of Petroleum (East China), Qingdao 266580, China; yanchangzhu2025@163.com (Y.Z.); taoyingyingupc@163.com (Y.T.); 13153398315@163.com (Q.W.); rui277940@163.com (R.Z.); xuechunyang61@gmail.com (Y.F.)

**Keywords:** nano-SiO_2_, steel fiber, polypropylene fiber, mechanical properties, Box–Behnken response surface design method, MOPSO algorithm, entropy weight TOPSIS decision method

## Abstract

With the increasing use of reinforced concrete segments in large-scale tunnels, engineering projects have placed higher mechanical demands on concrete, and the choice of concrete materials significantly influences these mechanical properties. This study is based on the preliminary mix design for the concrete used in the Second Undersea Tunnel Project, with the mass content of nano-SiO_2_ (NS) (1–3%), the volume content of steel fibers (SF) (0.5–1.5%) and the volume content of polypropylene fibers (PPF) (0.05–0.25%) as independent variables and using compressive strength (*Y*_1_), splitting tensile strength (*Y*_2_), and toughness index (*Y*_3_) as response variables. Using the Box–Behnken response surface design method, response surface models for each parameter were established and analyzed. The effects of NS, SF, and PPF on the mechanical properties of the concrete were investigated. Combining the MOPSO algorithm and the entropy-weighted TOPSIS method, a multi-objective cooperative optimization study was conducted. Finally, a microstructural analysis of the optimal NSDHFRC was performed. The results indicate that *Y*_1_, *Y*_2_, and *Y*_3_ all initially increase and then decrease with increasing NS content; *Y*_1_ and *Y*_3_ increase with increasing SF content. However, when the SF content exceeds a certain level, the fiber spacing becomes too dense, weakening the effective bridging effect between fibers, resulting in a decrease in *Y*_2_ at excessively high SF contents; PPF can suppress crack formation within a certain content range, but its effect on *Y*_1_ is relatively weak. Due to agglomeration and water absorption, both *Y*_2_ and *Y*_3_ decrease when the PPF content is too high. It was determined that the optimal solution occurs when the mass fraction of NS is 2.15%, and the volume fractions of SF and PPF are 1.37% and 0.063%, respectively, with *Y*_1_, *Y*_2_, and *Y*_3_ being 69.94 MPa, 5.49 MPa, and 1.99, respectively. Experimental verification confirmed that the relative error is within 5%. A microscopic analysis of the optimal solution revealed that an appropriate amount of NS refines the concrete structure through physical and chemical reactions, improves the interface transition zone, and enhances the bond strength between the fibers and the matrix. Meanwhile, PPF and SF distribute stress, respectively delaying the propagation of microcracks and macrocracks during different loading stages. These findings provide a reference for practical engineering applications.

## 1. Introduction

With the increasing use of reinforced concrete segments in large-scale tunnels, conventional concrete may be prone to premature cracking and crushing. Consequently, engineering projects now demand higher mechanical properties from concrete, making it crucial to enhance the mechanical properties of concrete used in tunnel lining segments.

Fiber reinforcement technology significantly enhances the toughness, crack resistance, and durability of concrete by forming a network structure [1]. Among various fibers, steel fibers and polypropylene fibers are most commonly used in concrete. Steel fibers, with their high elastic modulus and tensile strength, offer significant advantages in suppressing macrocrack propagation and enhancing concrete’s load-bearing capacity and ductility [2]. Polypropylene fibers primarily enhance concrete’s crack resistance, improving pore structure and delaying the initiation and propagation of microcracks [3]. This multi-scale reinforcement mechanism suggests that the combination of SF and PPF can provide crack control across different damage evolution stages: PPF inhibits microcrack formation, while SF controls macrocrack propagation and enhances ultimate load capacity. Wang et al. [4] conducted axial compression tests on cement composites reinforced with different ratios of steel fibers and polypropylene fibers. They found that as the ratio of steel fiber to polypropylene fiber increased, both the compressive strength and elastic modulus of the dual-fiber-reinforced cement composites rose. However, fibers primarily improve concrete’s internal structure through physical interactions. To meet high-strength requirements in concrete, current engineering practices commonly employ dual admixtures or chemical admixtures.

As one of the most effective novel materials, nanomaterials have significantly enhanced the mechanical properties of concrete [5]. Nano-SiO_2_ is the most popular zero-dimensional nanomaterial in engineering applications, exhibiting strong pozzolanic, nucleating, and micro-aggregate filling effects. These properties enhance the density of concrete structures, thereby improving their overall performance [6,7]. Previous studies on nano-SiO_2_ in cement-based materials can be categorized based on their research approaches. The first category focuses on the single incorporation of nano-SiO_2_. For instance, Nazari et al. [8] investigated the effect of replacing 0–5% nano-SiO_2_ with cement on the compressive strength of high-strength compacted concrete. They found that nano-SiO_2_ could enhance the flexural strength of concrete, but excessive nano-SiO_2_ would cause agglomeration and result in a decrease in strength. Similarly, Garg et al. [9] demonstrated that nanoscale silicates enhance compressive and tensile strength more effectively than microsilica, but reduce flowability. These studies established the fundamental benefits and limitations of nano-SiO_2_ alone. The second category explores the combination of nano-SiO_2_ with fibers. Mei et al. [10] investigated the effects of nano-SiO_2_ and different fibers (steel fibers and polypropylene fibers) on the mechanical properties of concrete, finding that their addition could enhance various mechanical properties. Emiru [11] indicated that a combination of 1.5% nano-silica and 1% silica fiber yields optimal performance. While these studies advanced understanding of binary combinations, they primarily reported performance enhancements without systematically analyzing the interactive effects between all components. The third category employs design optimization methodologies. Liu et al. [12] conducted a study on optimizing the mix design of polypropylene fibers using an orthogonal method, finding that fiber lengths between 9 and 21 mm positively enhance the mechanical properties of shotcrete. Prusty et al. [13] employed a four-factor, three-level orthogonal experimental design to optimize the mix proportions of concrete containing recycled aggregate and nano-SiO_2_. Preethi et al. [14] designed a three-factor, three-level orthogonal experiment with recycled asphalt, nano-clay, and nano-SiO_2_ as variables. More recently, Rahim et al. [15] employed response surface methodology to investigate the effects of rubber crumbs, fly ash, and nano-SiO_2_ on the workability and mechanical properties of high-strength self-compacting concrete, determining optimal proportions of 36.38% fly ash, 4.08% rubber crumb, and 1.0% nano-SiO_2_. Al-Sabaeei et al. [16] further advanced the field by combining response surface methodology with machine learning techniques to investigate waste denim fiber and nano-SiO_2_ composites in asphalt binders.

Despite these valuable contributions, a critical examination reveals that most existing studies either focus on single or binary material combinations, or employ optimization methods for different material systems. Systematic investigations that critically analyze the synergistic effects of nano-SiO_2_ when combined with both steel and polypropylene fibers in a ternary system remain limited. Furthermore, studies specifically targeting nano-SiO_2_-double-doped fiber high-strength concrete (NSDHFRC) using response surface methodology for multi-objective optimization are still scarce. Therefore, this study aims to address this gap by not only investigating the combined influence of NS, SF, and PPF but also by employing the Box–Behnken response surface design to quantitatively analyze and critically compare their interaction effects, thereby providing a robust foundation for mix design optimization [17,18,19]. Therefore, this study targets the optimization design of shield tunnel segment concrete for the Qingdao Jiaozhou Bay Second Subsea Tunnel Project, aiming to establish a scientifically based mix proportion that satisfies both mechanical performance and engineering applicability requirements under complex marine environments. Based on the preliminary concrete formulation design provided by the actual project, the content of nano-silicon oxygen, steel fiber and polypropylene fiber was selected as factors to make NSDHFRC. Using compressive strength, splitting tensile strength, and toughness index as response indicators, a response surface model (RSM) was established to investigate the interaction effects of NS, SF, and PPF on compressive strength, splitting tensile strength, and toughness index. By integrating the Multi-Objective Probability-Based Simultaneous Optimization (MOPSO) and Entropy-Weighted TOPSIS methods, this study optimizes the concrete mix design, providing a reference for its application and promotion in cross-sea tunnel engineering.

## 2. Materials and Methods

### 2.1. Materials

The cement used in this study was a 52.5 MPa ordinary Portland cement manufactured in Shandong Province. The fly ash used was grade I fly ash produced by Gongyi Borun Refractory Materials Co., Ltd., located in Gongyi, China. The mineral powder used was S95 grade mineral powder produced by Dehang Mineral Products Co., Ltd., located in Lingshou, China. The cement properties are shown in Table 1.

The NS product used in the trial was manufactured by Hebei Aosai Trading Co., Ltd. (Xingtai, China); the performance index of NS is shown in Table 2. SF and PPF used in this study were obtained from Hengshui Boge Metal Products Co., Ltd. (Hengshui, China), and Chuangsheng Building Materials Chemical Co., Ltd. (Shijiazhuang, China), and the steel fibers are hook-end type steel fibers. The basic performance indicators of SF and PPF are shown in Table 3 and Table 4, respectively.

The coarse aggregate used was 5~15 mm gravel, and the fine aggregate was coarse sand with fineness modulus of 2.6; both are supplied by the contractor. The water reducer was a high-performance water reducer produced by Hunan Zhongyan Building Materials Technology Co., Ltd. (Yueyang, China).

### 2.2. Test Scheme

This paper adopts the preliminary mix designs provided by the construction contractor of the Jiaozhou Bay Second Undersea Tunnel Project in accordance with JGJ 55-2011 [20] Specification for mix proportion design of ordinary concrete. The preliminary concrete mix designs are shown in Table 5.

In response surface experimental design, the Box–Behnken Design (BBD) and Central Composite Design (CCD) are commonly used methods. BBD is a spherical experimental design with rotational or quasi-rotational properties. Its characteristic is that the distances from each experimental point to the design center within the experimental region are essentially equal. This reduces the number of experiments required for the same factors and levels, allowing first- or second-order models of the relationship between factor variables and the response to be estimated using a relatively small number of experiments; CCD can better fit the response surface by setting extreme points, but the values of these extreme points may exceed the reasonable range of the factors, thereby affecting the reliability of the experimental results. Therefore, this study adopts the BBD method.

Using Design-Expert 13 software, a response surface design was conducted using the BBD method, with the mass fraction of NS (A), the volume fraction of SF (B), and the volume fraction of PPF (C) as factors. The response variables included the 28-day cube compressive strength (*Y*_1_), split tensile strength (*Y*_2_), and toughness index (*Y*_3_) of NSDHFRC. A total of 17 experiments were conducted, comprising 12 factorial experiments (C6–C17) and 5 replicate experiments at the center points of the design region (C1–C5). Three test specimens were prepared for each experimental group, and the results were averaged.

Following the methods described in [20,21,22,23,24], NS was used to replace 1%, 2%, and 3% of the cement; following the methods described in [25,26,27,28,29], the volume fractions of SF were set at 0.5%, 1%, and 1.5%, and those of PPF were set at 0.05%, 0.15%, and 0.25%. The test factors and level values are presented in Table 6; the mixing scheme of mixed fibers is shown in Table 7.

### 2.3. Specimen Preparation and Test Method

First, place the coarse aggregate, fine aggregate, and cementitious materials (cement, mineral powder, and fly ash) into the mixer and dry-mix for 60 s. To ensure thorough dispersion of the PPF, prepare a mixture of NS, water, and water-reducing agent, then incorporate the PPF into this mixture and mix in the mixer for 60 s. Finally, add the steel fibers to the mixer and mix for another 60 s. Pour the fully mixed slurry into standard test molds. After 1 day, demold the specimens and cure them in a constant-temperature chamber at 20 ± 2 °C and relative humidity greater than 95% for 28 days.

**Figure 1:**
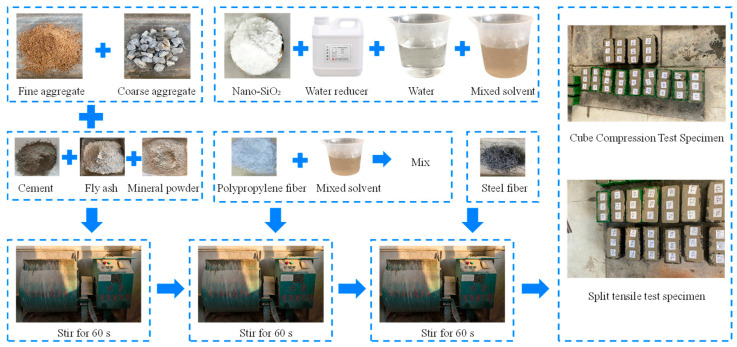
Concrete preparation process.

The dimensions of specimens used for both cube compression tests and splitting tensile tests were 100 mm × 100 mm × 100 mm, with three specimens per group. The specific procedure is illustrated in Figure 1.

**Figure 2:**
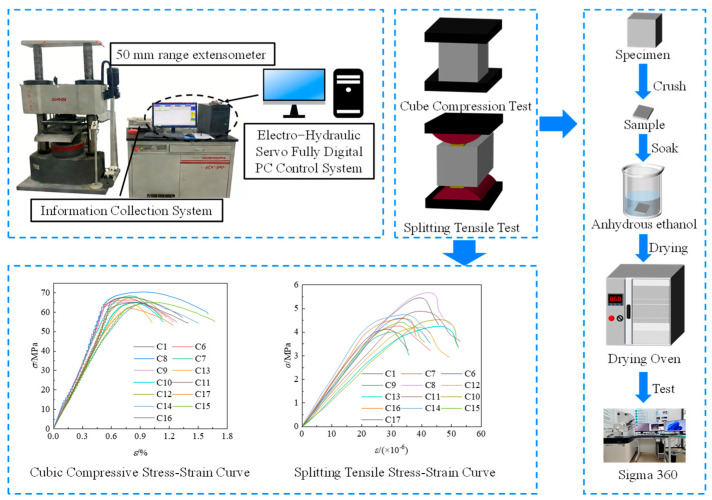
Static loading system.

In this paper, YAW6206 microcomputer-controlled electro-hydraulic servo pressure testing machine (Sans, Shenzhen, China) was used to carry out cube compression and splitting tests on the specimens in accordance with GB/T 50081-2019 Standard for Test Methods of Concrete Physical and Mechanical Properties [30]. The cube compressive strength reduction coefficient was 0.95, and the test was continuously and uniformly loaded at a rate of 0.5 MPa/s. The splitting tensile strength reduction coefficient was 0.85, and the test was continuously loaded at a rate of 0.2 MPa/s. Subsequently, a German ZEISS Sigma 360 field emission scanning electron microscope(SEM) was employed for microscopic analysis of both standard concrete and optimized concrete, which was provided by Hangzhou Yanqu Information Technology Co., Ltd. (Hangzhou, China). SEM observations of the microstructure of the concrete matrix and fibers facilitated the analysis of the effects of NS, SF, and PPF on the mechanical properties of the matrix. The static loading system is shown in Figure 2.

It is important to acknowledge that this study focused exclusively on the mechanical properties of hardened NSDHFRC. Therefore, workability measurements were not conducted. Similarly, fiber dispersion evaluation was not performed in this study, because the mechanical property results indirectly reflect the overall fiber dispersion quality.

## 3. Establishment and Discussion of Response Surface Model

### 3.1. Response Surface Model Establishment

Compressive toughness is a mechanical parameter used to quantitatively evaluate a material’s energy dissipation capacity under compression, reflecting concrete’s ability to absorb energy while continuing to bear loads after cracking [31]. Common methods include the energy method and the energy ratio method. Compared to the energy method, the energy ratio method distinguishes between the contributions of the matrix and fibers to compressive toughness [32]. The compressive toughness index *I* is as follows:

(1) $$I=\frac{U_{1}+U_{2}}{U_{1}}=\frac{\int_{0}^{\varepsilon_{cc,0.85}}\sigma dx}{\int_{0}^{\varepsilon_{cc}}\sigma dx}$$

where *I* represents the compressive toughness index calculated using the energy ratio method; *U*_1_ denotes the area enclosed by the stress–strain curve and the *x*-axis prior to the peak point; *U*_2_ denotes the area enclosed by the stress–strain curve and the *x*-axis between the peak point and the ultimate stress point.

The experimental results of cube compressive strength *Y*_1_, splitting tensile strength *Y*_2_ and toughness index *Y*_3_ of 28d NSDHFRC are shown in Table 8.

As shown in Table 8, the standard deviation of compressive strength ranges from 0.25 MPa to 0.36 MPa, the standard deviation of splitting tensile strength ranges from 0.206 MPa to 0.359 MPa, and the standard deviation of the toughness index ranges from 0.11 to 0.155. This indicates that the specimen preparation process was stable and that the test results exhibit a high degree of consistency.

Based on the test results, the prediction models of *Y*_1_, *Y*_2_ and *Y*_3_ on NS mass fraction (*A*), SF content (*B*) and PPF content (*C*) were established, and multiple regression analysis was carried out on these responses. The regression equations are shown in Equations (2)–(4). The prediction results are shown in Table 9.

(2) $$Y_{1}=48.65+10.25A+8.38B+35.47C+0.42AB-7.28AC-36.17BC-2.18A^{2}-0.87B^{2}+34.99C^{2}$$

(3) $$Y_{2}=-1.10+1.75A+6.58B+19.10C+0.33AB-0.01AC-4.55BC-0.48A^{2}-2.92B^{2}-50.98C^{2}$$

(4) $$Y_{3}=0.76+0.75A+0.79B+2.28C+0.01AB-0.74AC+0.121BC-0.192A^{2}-0.34B^{2}-4.88C^{2}$$

where *Y*_1_, *Y*_2_ and *Y*_3_ are the cube compressive strength (MPa), splitting tensile strength (MPa), and toughness index, respectively; *A*, *B* and *C* are the mass fraction of NS (%), volume fraction of SF (%), and volume fraction of PPF (%), respectively.

### 3.2. Response Surface Model Validation

The analysis of variance (ANOVA) and reliability analysis on the established model to evaluate the accuracy of the response surface regression model. ANOVA for *Y*_1_, *Y*_2_, and *Y*_3_ are presented in Table 10, Table 11 and Table 12, respectively. Additionally, the statistical measure *F*-value must be considered, which is the ratio of the mean square of the regression to the mean square of the residuals. Its calculation formula is

(5) $$F=\frac{M_{r}}{M_{e}}=\frac{S_{r}/v}{S_{e}/(n-v-1)}$$

where *M*_r_ denotes the mean square of regression; *M*_e_ denotes the mean square of residuals; *S*_r_ denotes the sum of squares of regression; *S*_e_ denotes the sum of squares of residuals; *n* denotes the number of experimental groups; and *v* denotes the number of response model variables.

A larger *F*-value corresponds to a smaller *p*, which represents the probability that the *F* is less than the critical value *F*_0_; The significance of the model can be determined based on the *p*. When *p* < 0.01, the model is considered highly significant; when 0.01 ≤ *p* ≤ 0.05, it is considered significant; and when *p* > 0.05, it is deemed not significant.

According to Table 10, Table 11 and Table 12, the VIF values for all terms in the three response surface models are close to 1, indicating that there is no multicollinearity in the models. This confirms that the estimated coefficients are reliable and that the effects of the factors can be interpreted independently. Statistical significance was assessed at a 95% confidence level (*p* < 0.05). As shown by the ANOVA results, all models exhibited high statistical significance. In the cube compressive strength model, the significant terms included *A*, *B*, *C*, *AC*, *BC*, and *A*^2^; in the split tensile strength model, the significant terms included *A*, *B*, *AB*, *BC*, *A*^2^, *B*^2^, and *C*^2^; in the toughness index model, significant terms included *A*, *B*, *C*, *AC*, *A*^2^, *B*^2^, and *C*^2^. Furthermore, the *p*-values for the lack of fit value in each model were greater than 0.05, indicating that the models fit the data well and enhancing their reliability.

The coefficient of determination *R*^2^ is one of the key indicators for assessing model quality. *R*^2^ evaluates the degree of fit between the model and the empirical data, ranging from 0 to 1; the closer *R*^2^ is to 1, the higher the degree of fit. The coefficients of determination *R*^2^ for these three models and other model validation results are shown in Table 13. It can be seen that the *R*^2^ values for *Y*_1_, *Y*_2_, and *Y*_3_ are all close to 1, at 0.9628, 0.9820, and 0.9763, respectively. Furthermore, a coefficient of variation (C.V.) below 10% and a signal-to-noise ratio above 4 are preferred. As shown in Table 13, the coefficients of variation for *Y*_1_, *Y*_2_, and *Y*_3_ are 18.2494%, 18.2494%, and 18.2494%, respectively, while the signal-to-noise ratios are 0.81, 2.57, and 2.03, respectively. These values indicate that the model is sound and can be used for response prediction.

**Figure 3:**
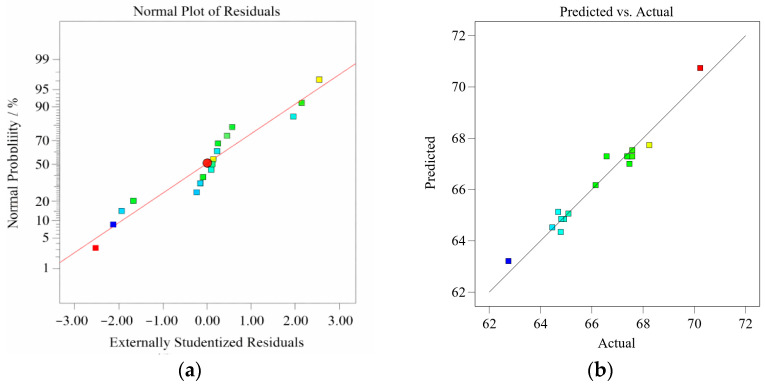
(**a**) Normal plot of residuals and (**b**) predicted versus actual plot for *Y*_1_.

**Figure 4:**
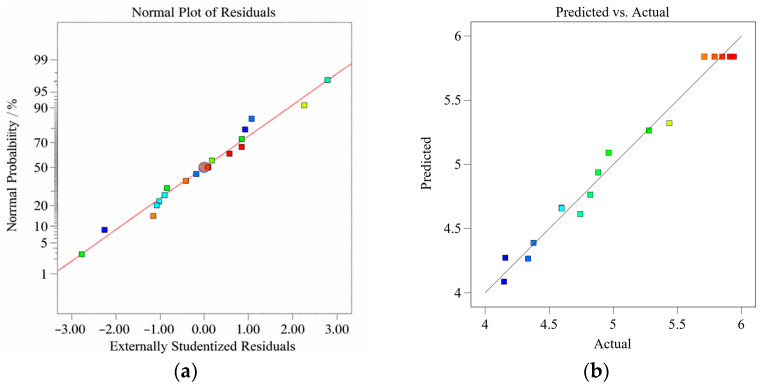
(**a**) Normal plot of residuals and (**b**) predicted versus actual plot for *Y*_2_.

**Figure 5:**
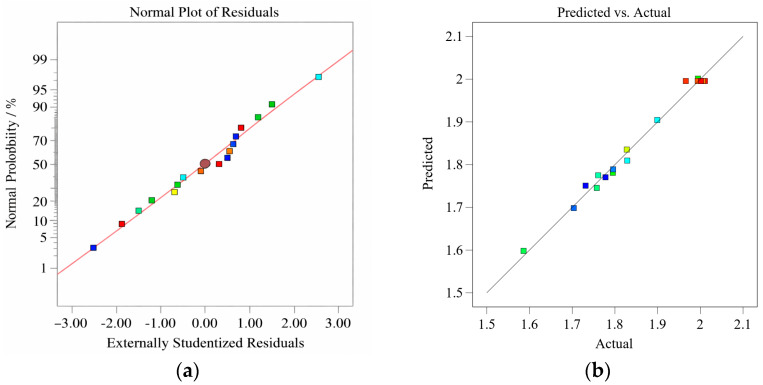
(**a**) Normal plot of residuals and (**b**) predicted versus actual plot for *Y*_3_.

To further evaluate the applicability of the established *Y*_1_, *Y*_2_, and Y_3_ response surface models, we analyzed the normal probability plots of the residuals and the comparison plots of measured and predicted values, as shown in Figure 3, Figure 4 and Figure 5. There is a significant linear correlation between the observed and predicted values, indicating that all three models exhibit satisfactory predictive performance. Furthermore, the linear trend in the normal probability plot suggests that the residuals follow an approximately normal distribution. This conclusion is supported by the fact that approximately 95% of the standardized residuals fall within the range of ±2%, satisfying the normality assumption of regression analysis [33].

In summary, all three predictive models accurately describe the functional relationship between the target quantity and design variables, and can be utilized for subsequent response surface analysis and mix design optimization of NSDHFRC.

### 3.3. Response Surface Analysis

#### 3.3.1. Analysis of Cube Compressive Strength

**Figure 6:**
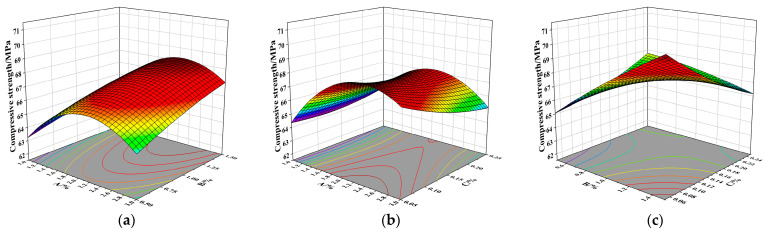
Response surface diagrams of compressive strength for NSDHFRC. (**a**) Interaction between NS and SF. (**b**) Interaction between NS and PPF. (**c**) Interaction between SF and PPF.

As shown in the response surface plot Figure 6 and Table 10, the model exhibits a high F-value of 20.10, indicating that it is statistically significant. The probability that such a large F-value is due to noise is only 0.03%. Figure 6a shows that the interaction between NS content and SF content (denoted as AB) is not particularly significant. When the PPF volume content is 0.15%, the compressive strength of the specimens first increases and then decreases with increasing NS content; this is due to the pozzolanic activity and filling effect of NS; Appropriate amounts of NS can optimize the matrix structure, whereas excessive amounts lead to agglomeration, thereby weakening the pozzolanic activity and hindering chemical reactions, which in turn reduces the pozzolanic activity [34]; conversely, as the SF content increases, the compressive strength shows a steady upward trend. This is because SF acts as a bridging agent; under fixed PPF content conditions, it enhances the density of the concrete, thereby improving the compressive strength.

As shown in Figure 6b, there is a strong interaction between NS and PPF on compressive strength, and the NS content has a significant effect on compressive strength. When the volume content of SF is 1%, the compressive strength of the concrete first increases and then decreases with increasing NS content; initially, the compressive strength decreases with increasing PPF content. This is because PPF tends to absorb free water in the mixture, and an excessive amount of PPF can hinder the secondary hydration of NS, leading to increased porosity and reduced strength [35].

As shown in Figure 6c, SF has a greater influence on compressive strength than PPF. When the SF content is below 1%, the compressive strength of concrete increases gradually with increasing PPF dosage. However, once the SF content exceeds 1%, the compressive strength begins to decrease as PPF content increases. Similarly, when the PPF content is below 0.2%, the compressive strength increases with the SF content, but beyond this PPF threshold, the compressive strength shows little variation or even a slight decline with further SF addition.

#### 3.3.2. Analysis of Splitting Tensile Strength

**Figure 7:**
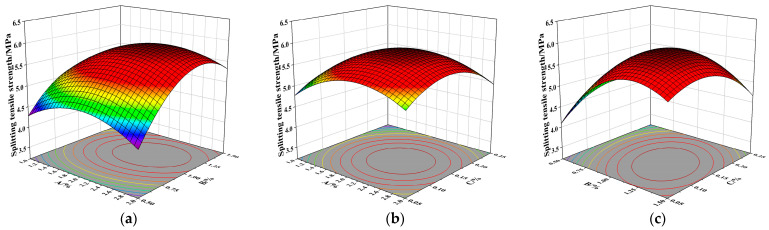
Response surface diagrams of splitting tensile strength for NSDHFRC. (**a**) Interaction between NS and SF. (**b**) Interaction between NS and PPF. (**c**) Interaction between SF and PPF.

As shown in Figure 7a, the response surface exhibits high curvature and elliptical contour lines, indicating that the interaction between NS and SF significantly influences the splitting tensile strength of concrete. At a PPF volume content of 0.15%, the splitting tensile strength of the concrete first increases and then decreases with increasing NS content, and the final arch curve approaches a power–law curve, which is similar to the mechanism observed for compressive strength; The splitting tensile strength first increases and then decreases with increasing SF content. This is because splitting tensile strength primarily reflects the concrete’s ability to resist crack formation and propagation under radial tensile stress. When the SF content exceeds a certain level, the fibers become too close together, causing the effective bridging effect between fibers to decrease, thereby reducing the reinforcement effectiveness for concrete with only a single main crack [36]. The density of contour lines on the vertical axis is higher than that on the horizontal axis, indicating that the effect of SF content on split tensile strength is more significant.

As shown in Figure 7b, the contour lines appear approximately circular, indicating that the interaction between NS and PPF has virtually no effect on the splitting tensile strength. Furthermore, the density of contour lines on both the vertical and horizontal axes is similar, suggesting that these two factors exert a comparable influence on the splitting tensile strength. When the volume content of SF is 1%, the splitting tensile strength first increases and then decreases with increasing NS and PPF. This may be because excess PPF aggregates and absorbs free water, thereby inhibiting the secondary hydration of NS, while excess NS also tends to agglomerate, resulting in incomplete hydration and a reduction in splitting strength.

As shown in Figure 7c, The interaction between SF and PPF significantly affects splitting tensile strength, with contour lines denser along the horizontal axis than the vertical axis, indicating that SF dosage exerts a greater influence on splitting tensile strength. When SF dosage is approximately 1.25% and PPF dosage is approximately 0.15%, splitting tensile strength exhibits a maximum value within a certain range. The combination of SF and PPF can produce a positive mixing effect, enhancing the splitting tensile strength of concrete. However, when the fiber content is too high, the fibers tend to agglomerate, thereby weakening the synergistic toughening effect.

#### 3.3.3. Analysis of Toughness Index

**Figure 8:**
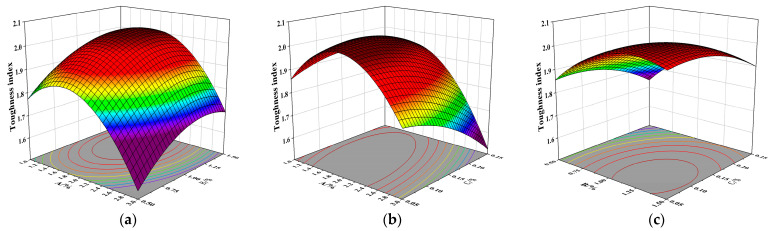
Response surface diagrams of toughness index for NSDHFRC. (**a**) Interaction between NS and SF. (**b**) Interaction between NS and PPF. (**c**) Interaction between SF and PPF.

As shown in Figure 8a, the contour lines exhibit an elliptical shape. The interaction between NS and SF significantly influences the toughness index, with the horizontal axis of the contour lines being denser than the vertical axis. This indicates that the NS dosage exerts a greater impact on the toughness index. And the toughness index increases with increasing SF content.

Figure 8b shows that the toughness index first increases and then decreases with increasing NS content, and gradually increases with increasing SF content. The toughness index reaches its minimum value when the NS content is 3% and the PPF content is 0.5%. This is due to the redundancy of NS: while part of it fills the voids and bonds the fibers, the excess forms agglomerates, leading to local inhomogeneity in the concrete. The maximum value of the toughness index occurs within the range of 1.6–1.8% NS content and 0.05–0.1% PPF content. Furthermore, it can be observed that the interaction between NS and PPF significantly influences the toughness index. The density of contour lines along the vertical axis is higher than that along the horizontal axis, which again indicates that NS has a greater influence on the toughness index.

In Figure 8c, the contour lines also display an elliptical shape, signifying a significant interaction between SF and PPF. The higher contour density along the horizontal axis indicates that SF content plays a more dominant role in influencing the toughness index.

## 4. Multi-Objective Mix Design Optimization Based on MOPSO

### 4.1. Optimization Model Construction

The optimization of concrete mix proportions inherently involves multiple conflicting performance objectives. In this study, three key mechanical properties—*Y*_1_, *Y*_2_, and *Y*_3_—must be simultaneously considered. These objectives exhibit trade-off relationships, improving one property often comes at the expense of others; therefore, a multi-objective optimization approach is necessary to generate a set of Pareto-optimal solutions that explicitly represent the trade-offs among competing objectives. Compared with evolutionary algorithms such as NSGA-II and traditional genetic algorithms, MOPSO requires fewer control parameters and does not rely on complex genetic operators such as crossover and mutation; consequently, it is simpler to implement and incurs lower computational costs. Furthermore, it demonstrates a relatively fast convergence rate in continuous multi-objective optimization problems. Given that the design variables in this study (NS, SF and PPF content) are continuous mixture ratios rather than discrete variables, MOPSO is particularly well-suited for efficiently approximating a well-distributed Pareto front in medium-scale engineering optimization tasks [37]. Consequently, MOPSO was selected as the optimization tool in this study to ensure computational efficiency.

MOPSO is a population-based optimization algorithm inspired by the social behavior of bird flocks or fish schools (particles). Each particle represents a potential solution and updates its position and velocity by tracking its personal best (*PBest*) and the global best (*GBest*) solutions, effectively guiding the population to converge toward the Pareto front. The specific update process is shown in Equations (6) and (7).

(6) $$x_{i}^{t+1}=x_{i}^{t}+v_{i}^{t+1}$$

(7) $$v_{i}^{t+1}=\omega v_{i}^{t}+c_{1}\gamma_{1}(PBest_{i}^{t}-x_{i}^{t})+c_{2}\gamma_{2}(GBest_{i}^{t}-x_{i}^{t})$$

where $x_{i}^{t}$ denotes the position vector of particle *i* at iteration *t*; $v_{i}^{t}$ is the velocity vector of particle *i* at iteration *t*; *ω* represents the inertia weight factor; *c*_1_ and *c*_2_ are learning coefficients that regulate the cognitive weights of individual and social experiences, respectively; *γ*_1_ and *γ*_2_ are random variables uniformly distributed over the interval [0, 1]; ${PBest}_{i}^{t}$ is the personal best position of particle *i* up to iteration *t*; and ${GBest}_{i}^{t}$ denotes the global best position.

Using response surface models *Y*_1_, *Y*_2_ and *Y*_3_ as performance objective functions, the optimization of NSDHFRC can be formulated as follows:

(8) $$\max Y_{i}=RSM(x_{1},x_{2},x_{3})$$

where *Y_i_* represents the response surface prediction models *Y*_1_, *Y*_2_, *Y*_3_; *x*_1_, *x*_2_ and *x*_3_ denote the dosages of NS, SF, and PPF, respectively.

In this study, all objective functions are formulated as maximization problems, with compressive strength, splitting tensile strength, and toughness index set as the performance objectives. The corresponding objective function models are constructed as follows:

(9) $$\min\left\{-Y_{1},-Y_{2},-Y_{3}\right\}$$

The constraints of the model are defined as follows: the mass fraction of NS (*x*_1_) should be between 1% and 3%, the volume fraction of SF (*x*_2_) between 0.5% and 1.5%, and the volume fraction of PPF (*x*_3_) between 0.05% and 0.25%.

In this study, multi-objective optimization of the NSDHFRC mix proportions was conducted using the MOPSO algorithm implemented in MATLAB R2024b. After extensive simulations, the final parameter settings are summarized in Table 14.

### 4.2. Optimization Results and Analysis

**Figure 9:**
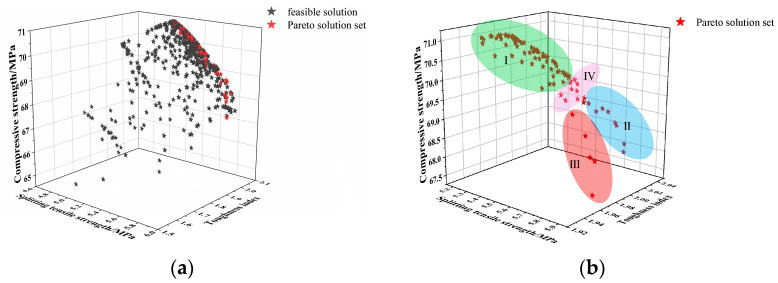
Pareto front and non-dominated solution sets for different objective regions. (**a**) Pareto front; (**b**) non-dominated solution sets for different objective regions.

Based on the established objective function, constraints, and corresponding parameters, the MOPSO model was employed to perform global optimization of the mix design. The resulting Pareto front is illustrated in Figure 9.

As shown in Figure 9, the Pareto front curve is smooth and presents a uniformly distributed surface, effectively dominating other solutions, which indicates good optimization performance. The distribution of the solution set reveals that achieving a high level in one performance metric of NSDHFRC generally comes at the expense of other metrics. Different optimization schemes are therefore adopted according to varying design objectives. The division of the non-dominated solutions into Regions I–IV in Figure 9b is intended to serve as a practical reference for engineering applications. The boundaries of these regions are illustrative. For applications requiring very high compressive strength, solutions from Region I are recommended; for high toughness index demands, non-dominated solutions in Region II are preferable; for elevated splitting tensile strength requirements, non-dominated solutions in Region III are suitable. To obtain NSDHFRC with excellent comprehensive performance, non-dominant solutions from Region IV may be selected.

### 4.3. Optimal Pareto Solution Based on Entropy Weight TOPSIS Decision-Making

Although MOPSO generates a set of Pareto-optimal solutions, a decision-making method is still required to select the most appropriate solution from among them based on engineering priorities. The Entropy-Weighted TOPSIS method quantifies the differences in importance between criteria using information entropy, based on the dispersion of objective function values within the set of Pareto-optimal solutions. A smaller entropy value indicates greater variability in the data distribution of that objective within the solution set, implying a higher weight for that criterion and, consequently, stronger discriminatory power in the decision-making process. After constructing a normalized decision matrix, the Euclidean distances between each Pareto solution and the positive and negative ideal solutions are calculated. By dynamically generating a reference framework of ideal solutions, this method overcomes the limitations of fixed thresholds when adapting to complex Pareto fronts. The calculation method is as follows:

Prepare the original matrix.

That is, the scoring data of different objects under various indicators, represented as an *n* × *m* matrix, where *n* is the number of objects and *m* is the number of indicators.

(10) $$X=\left[\begin{array}{llll} x_{11} & x_{12} & \ldots & x_{1m}\\ x_{21} & x_{22} & \ldots & x_{2m}\\ \vdots & \vdots & \ddots & \vdots \\ x_{n1} & x_{n2} & \ldots & x_{nm} \end{array}\right]$$

where *X* is the original evaluation matrix; *x*_ij_ denotes the value of the *j*-th evaluation criterion in the *i*-th mix ratio optimization scheme, where *i* ∈ [1, *n*], *j* ∈ [1, *m*], *n* is the number of optimization schemes, and m is the number of evaluation criteria.

The range method is used to normalize and positively orient the original matrix, where all performance objective functions in this study are considered as beneficial indicators. The normalization formula and the resulting normalized matrix are as follows:

(11) $$z_{ij}=\frac{x_{ij}-x_{j}^{\min}}{x_{j}^{\max}-x_{j}^{\min}}$$

(12) $$Z=\left[\begin{array}{llll} z_{11} & z_{12} & \ldots & z_{1m}\\ z_{21} & z_{22} & \ldots & z_{2m}\\ \vdots & \vdots & \ddots & \vdots \\ z_{n1} & z_{n2} & \ldots & z_{nm} \end{array}\right]$$

Calculate the proportion *P_ij_* of the *i*-th object under the *j*-th indicator.

(13) $$P_{ij}=z_{ij}/\sum_{i=1}^{n}z_{ij},\left(j=1,2,\ldots ,m\right)$$

Calculate the entropy weight *w_j_* of the *j*-th indicator.

(14) $$w_{j}=\frac{1-E_{j}}{\sum_{j=1}^{m}1-E_{j}}$$

(15) $$E_{j}=-\frac{1}{\ln n}\sum_{i=1}^{n}P_{ij}\ln P_{ij},\left(j=1,2,\ldots ,m\right)$$

where *E_j_* is the entropy value of the *j*-th evaluation indicator. When *P_ij_* = 0, the term *P_ij_*ln*P_ij_* = 0.

The computed weights of each evaluation indicator are presented in Table 15.

Construct the weighted matrix *C*.

(16) $$c_{ij}=w_{j}z_{ij}$$

(17) $$C=\left[\begin{array}{llll} c_{11} & c_{12} & \ldots & c_{1m}\\ c_{21} & c_{22} & \ldots & c_{2m}\\ \vdots & \vdots & \ddots & \vdots \\ c_{n1} & c_{n2} & \ldots & c_{nm} \end{array}\right]$$

Calculate the positive *C*^+^ and negative *C*^−^ ideal solutions.

(18) $$C^{+}=(C_{1}^{+},C_{2}^{+},\ldots ,C_{m}^{+})$$

(19) $$C^{-}=(C_{1}^{-},C_{2}^{-},\ldots ,C_{m}^{-})$$

Calculate the Euclidean distances between each object and the positive and negative ideal solutions $D_{i}^{+}$ and $D_{i}^{-}$.

(20) $$D_{i}^{+}=\sqrt{\sum_{j=1}^{m}{\left(C_{j}^{+}-c_{ij}\right)}^{2}}$$

(21) $$D_{i}^{-}=\sqrt{\sum_{j=1}^{m}{\left(C_{j}^{-}-c_{ij}\right)}^{2}}$$

Determine the closeness coefficient *O_i_* of each mix proportion optimization scheme to the ideal solutions. The closer *O_i_* is to 1, the better the evaluation performance of the scheme.

(22) $$O_{i}=\frac{D_{i}^{-}}{D_{i}^{+}+D_{i}^{-}}$$

**Figure 10:**
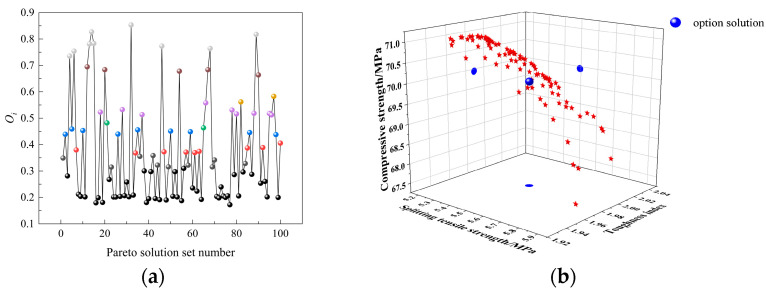
Relative closeness curve and optimal solution. (**a**) Relative closeness curve. (**b**) Optimal solution.

The relative proximity index *O_i_* of each scheme on the Pareto frontier to the ideal solution was calculated. *O_i_* comprehensively considers the compressive strength, splitting tensile strength, and toughness index of NSDHFRC, objectively reflecting the degree of closeness between each evaluated scheme and the ideal solution. The closer *O_i_* is to 1, the better the evaluation result of the scheme. The results are shown in Figure 10. As shown in Figure 10a, Scheme 32 exhibits the highest relative proximity, being closer to the ideal solution (see Figure 10b). This indicates that Scheme 32 possesses the optimal comprehensive performance of NSDHFRC; thus, it is determined as the optimal mix design. The final optimized results are: NS volume content of 2.15%, SF volume content of 1.37%, and PPF volume content of 0.063%.

This design was experimentally verified, with the obtained performance indicators listed in Table 16. As shown in Table 16, all experimental values fall within the 95% prediction intervals for each parameter, validating the effectiveness and reliability of the RSM coupled with MOPSO and entropy-weighted TOPSIS method for the multi-objective optimization design of NSDHFRC mix proportions.

### 4.4. SEM Analysis

**Figure 11:**
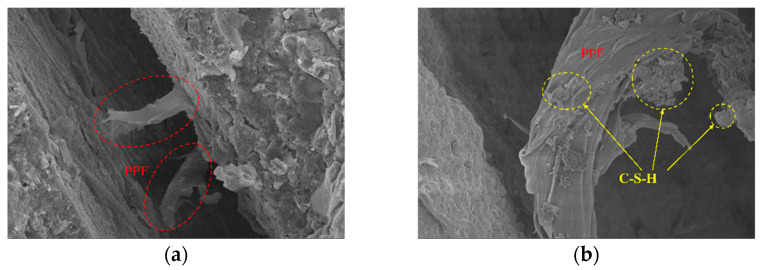

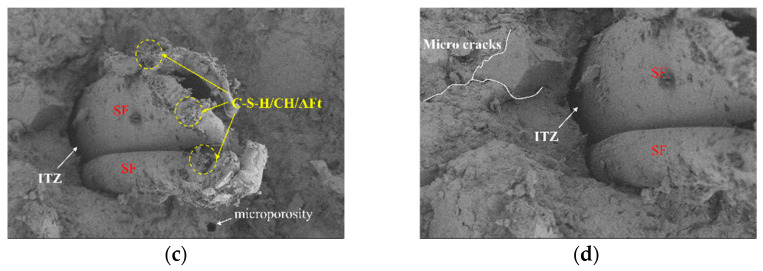
Microstructure of fibers for optimal NSDHFRC. (**a**) PPF snapped and magnified one thousand times; (**b**) PPF snapped and magnified five thousand times; (**c**) SF snapped and magnified three hundred times; (**d**) SF snapped and magnified five hundred times.

To further investigate the reinforcement effects of NS, SF, and PPF on concrete, SEM analysis was conducted on ordinary concrete and the optimized NSDHFRC. Figure 11 shows SEM images of the NSDHFRC after crushing.

As shown in Figure 11a,b, PPF mainly exhibits tensile fracture with a relatively smooth surface and limited plastic deformation. The presence of C–S–H gel adhered to the PPF surface indicates that chemical bonding and mechanical interlocking exist between the fiber and the cement matrix. This C–S–H is likely generated by the pozzolanic reaction between NS and Ca(OH)_2_ in the cement matrix, which refines the interfacial microstructure. However, due to the relatively low elastic modulus of PPF, the fiber primarily contributes through elastic deformation and microcrack bridging at the early stage of crack development. Once the crack opening exceeds the elastic deformation capacity of PPF, fiber rupture occurs rapidly, explaining the limited energy dissipation capacity observed at later stages.

In contrast, as shown in Figure 11c,d, SF exhibit tensile fracture with rougher surfaces and signs of plastic deformation. Hydration products, including C–S–H and AFt, are clearly observed on the SF surface and within the interfacial transition zone (ITZ). This suggests that NS modification promotes a denser ITZ by consuming Ca(OH)_2_ and generating additional C–S–H gel, thereby improving fiber–matrix bond strength. The enhanced ITZ reduces interfacial micro-porosity and increases frictional resistance during fiber pull-out. As a result, SF can sustain higher loads and undergo significant deformation before failure, contributing effectively to crack bridging and energy dissipation during crack propagation.

Overall, the synergistic interaction among NS, fibers, and the cement matrix promotes hydration progression and microstructural densification. The formation of a composite interfacial structure consisting of fibers, hydration products, and a refined ITZ significantly enhances fiber–matrix bonding. This improved interfacial integrity enables fibers to actively participate throughout the crack evolution process, thereby enhancing tensile performance and structural stability.

## 5. Conclusions

(1) A multivariate regression model based on the Box–Behnken response surface design was developed to quantitatively describe the effects and interactions of the three factors NS, SF, and PPF. Analysis of variance and signal-to-noise ratio tests showed that the coefficients of determination (*R*^2^) is close to 1, the C.V. is less than 10%, and the signal-to-noise ratio is consistently above 4, indicating that the established predictive models possess excellent fitting accuracy.
(2) NS, SF, and PPF each have significant influences on the mechanical properties of the concrete. The results indicate that cubic compressive strength, split tensile strength, and resilience index all initially increase and then decrease with increasing NS content; cubic compressive strength and resilience index increase with increasing SF content. However, when the SF content exceeds a certain level, the fiber spacing becomes too dense, weakening the effective bridging effect between fibers, resulting in a decrease in split tensile strength at excessively high SF contents; PPF can suppress crack formation within a certain content range, but its effect on cubic compressive strength is relatively weak; Due to agglomeration and water absorption, both split tensile strength and resilience index decrease when the PPF content is too high.
(3) The mix proportion of NSDHFRC was optimized through a multi-objective approach combining MOPSO and entropy-weighted TOPSIS methods. The optimal mix was determined to be the mass fraction of NS is 2.15%, and the volume fractions of SF and PPF are 1.37% and 0.063%, respectively, with cubic compressive strength, split tensile strength, and resilience index being 69.94 MPa, 5.49 MPa, and 1.99, respectively. Experimental verification confirmed that the relative error is within 5%, validating the effectiveness and practical applicability of the coupled response surface methodology and multi-objective optimization techniques for NSDHFRC mix design, providing a basis and reference for practical engineering projects.
(4) Compared to ordinary concrete, the incorporation of NS in NSDHFRC effectively promotes hydration reactions, generating abundant C-S-H gel and resulting in a denser matrix structure. Simultaneously, under the modifying effect of NS, both PPF and SF surfaces are coated with substantial hydration products, forming a stable “fiber-hydration product-matrix” composite interface structure that significantly enhances interfacial bonding between fibers and matrix. PPF primarily acts during the microcrack initiation stage, inhibiting early damage development, while SF continuously provides bridging and load-bearing functions during crack propagation and post-peak stages.

## Figures and Tables

**Table 1:** Basic performance indicators of P·O 52.5 cement.

28-Day Compressive Strength/MPa	Initial Setting Time/h	Setting Time/h	Water-to-Clinker Ratio/%	Moisture Content/%	0.08 mm Sieve Size/%	CaO/%	SiO_2_/%
53	50	8	84	99.9	0.005	54.82	23.89

**Table 2:** Chemical composition and physical performance indicators of nano-SiO_2_.

Appearance	Bulk Density /(g·cm^−3^)	Specific Surface Area/(m^2^·g)	Average Particle Size/nm	SiO_2_ /%	Fe_2_O_3_ /%	Al_2_O_3_ /%
White powder	0.15	185	20	99.9	0.005	0.04

**Table 3:** Steel fiber basic performance indicators.

Density /(g·cm^−3^)	Diameter /mm	Length /mm	Diameter-to-Length Ratio	Tensile Strength /MPa
7.8	0.75	35	47	1150

**Table 4:** Polypropylene fiber basic performance indicators.

Density /(g·cm^−3^)	Melting Point/°C	Diameter /μm	Length /mm	Tensile Strength /MPa	Modulus of Elasticity/GPa
0.91	167	26	18	510	5.5

**Table 5:** Preliminary design of concrete mix proportions (kg/m^3^).

Cement	Fly Ash	Mineral Powder	Aggregate		Water	Water Reducer
			Coarse Aggregate	Fine Aggregate		
321	50	124	1181	665	131	5.93

**Table 6:** Factors and level of experiment.

Factors	Number	Level		
		−1	0	1
NS	A/%	1	2	3
SF	B/%	0.5	1	1.5
PPF	C/%	0.05	0.15	0.25

**Table 7:** The mixing scheme of mixed fibers.

Test Number	Factors		
	A/%	B/%	C/%
C1	2	1	0.15
C2	2	1	0.15
C3	2	1	0.15
C4	2	1	0.15
C5	2	1	0.15
C6	2	1.5	0.25
C7	2	0.5	0.25
C8	2	1.5	0.05
C9	2	0.5	0.05
C10	3	1	0.25
C11	3	1	0.05
C12	3	1.5	0.15
C13	3	0.5	0.15
C14	1	1	0.25
C15	1	1	0.05
C16	1	1.5	0.15
C17	1	0.5	0.15

**Table 8:** Test results and standard deviation.

Number	*Y*_1_/MPa			*Y*_2_/MPa			*Y*_3_		
	Result	Mean	Sd	Result	Mean	Sd	Result	Mean	Sd
C1	67.05, 67.32, 67.77	67.38	0.36	5.70, 6.05, 6.07	5.94	0.206	1.95, 2.09, 2.11	2.05	0.084
C2	67.31, 67.62, 67.81	67.58	0.25	5.40, 5.81, 5.92	5.71	0.271	1.96, 1.97, 2.10	2.01	0.078
C3	67.18, 67.48, 67.90	67.52	0.36	5.60, 5.95, 6.18	5.91	0.29	1.93, 1.99, 2.05	1.99	0.06
C4	66.29, 66.61, 66.84	66.58	0.28	5.50, 5.92, 6.13	5.85	0.322	1.92, 2.03, 2.05	2	0.071
C5	67.11, 67.45, 67.70	67.42	0.30	5.42, 5.88, 6.07	5.79	0.334	1.90, 2.01, 2.03	1.98	0.07
C6	65.88, 66.09, 66.48	66.15	0.3	4.25, 4.70, 4.85	4.6	0.312	1.81, 1.92, 1.97	1.9	0.081
C7	67.95, 68.18, 68.59	68.24	0.33	4.05, 4.48, 4.61	4.38	0.292	1.68, 1.75, 1.76	1.73	0.044
C8	69.98, 70.15, 70.56	70.23	0.30	4.95, 5.40, 5.49	5.28	0.291	1.89, 2.05, 2.06	2	0.094
C9	64.82, 65.03, 65.42	65.09	0.30	3.80, 4.25, 4.40	4.15	0.315	1.81, 1.85, 1.92	1.86	0.056
C10	64.41, 64.73, 64.93	64.69	0.26	4.55, 5.00, 5.09	4.88	0.288	1.51, 1.58, 1.59	1.56	0.044
C11	67.28, 67.52, 67.94	67.58	0.33	4.60, 5.10, 5.18	4.96	0.315	1.76, 1.83, 1.84	1.81	0.044
C12	67.15, 67.50, 67.76	67.47	0.31	5.10, 5.55, 5.67	5.44	0.302	1.61, 1.65, 1.72	1.66	0.056
C13	64.18, 64.40, 64.80	64.46	0.31	4.00, 4.45, 4.57	4.34	0.303	1.48, 1.48, 1.57	1.51	0.052
C14	64.52, 64.85, 65.06	64.81	0.27	4.40, 4.85, 4.97	4.74	0.303	1.85, 1.87, 1.89	1.87	0.02
C15	64.48, 64.83, 65.06	64.79	0.29	4.48, 4.95, 5.03	4.82	0.311	1.75, 1.80, 1.91	1.82	0.08
C16	64.63, 64.88, 65.25	64.92	0.31	4.20, 4.75, 4.85	4.6	0.351	1.89, 1.94, 1.99	1.94	0.05
C17	62.48, 62.79, 62.98	62.75	0.25	3.75, 4.30, 4.43	4.16	0.359	1.76, 1.78, 1.86	1.8	0.053

**Table 9:** Experimental and predicted results.

Number	*Y*_1_		*Y*_2_		*Y*_3_	
	Actual Value	Predicted Value	Actual Value	Predicted Value	Actual Value	Predicted Value
C1	67.38	67.3	5.94	5.84	2.05	2.00
C2	67.58	67.3	5.71	5.84	2.01	2.00
C3	67.52	67.3	5.91	5.84	1.99	2.00
C4	66.58	67.3	5.85	5.84	2	2.00
C5	67.42	67.3	5.79	5.84	1.98	2.00
C6	66.15	66.17	4.6	4.66	1.9	1.9
C7	68.24	67.73	4.38	4.39	1.73	1.74
C8	70.23	70.74	5.28	5.26	2.00	1.99
C9	65.09	65.07	4.15	4.09	1.86	1.85
C10	64.69	65.13	4.88	4.94	1.56	1.52
C11	67.58	67.54	4.96	5.09	1.81	1.78
C12	67.47	67.01	5.44	5.32	1.66	1.7
C13	64.46	64.53	4.34	4.27	1.51	1.53
C14	64.81	64.85	4.74	4.61	1.87	1.9
C15	64.79	64.35	4.82	4.76	1.82	1.86
C16	64.92	64.85	4.6	4.66	1.94	1.91
C17	62.75	63.22	4.16	4.27	1.8	1.77

**Table 10:** ANOVA results for *Y*_1_.

Source	Sum of Squares	df	Mean Square	F-Value	*p*-Value	VIF	Significance
*Y*_1_	52.2844	9	5.8094	20.1031	0.0003	—	Yes
*A*-NS	6.0031	1	6.0031	20.7735	0.0026	1	Yes
*B*-SF	8.4598	1	8.4598	29.2748	0.0010	1	Yes
*C*-PPF	1.8019	1	1.8019	6.2353	0.0412	1	Yes
*AB*	0.1764	1	0.1764	0.6104	0.4602	1	No
*AC*	2.117	1	2.117	7.3259	0.0303	1	Yes
*BC*	13.0801	1	13.0802	45.2633	0.0003	1	Yes
*A* ^2^	19.981	1	19.981	69.1433	<0.0001	1.01	Yes
*B* ^2^	0.1993	1	0.1993	0.6898	0.4336	1.01	No
*C* ^2^	0.5155	1	0.5155	1.784	0.2235	1.01	No
Residual	2.0229	7	0.289	—	—	—	—
Lack of fit	1.357	3	0.4523	2.7169	0.1793	—	No
Pure Error	0.6659	4	0.1665	—	—	—	—
Cor Total	54.3074	16	—	—	—	—	—

**Table 11:** ANOVA results for *Y*_2_.

Source	Sum of Squares	df	Mean Square	F-Value	*p*-Value	VIF	Significance
*Y*_2_	6.4	9	0.7114	42.41	<0.0001	—	Yes
*A*-NS	0.2115	1	0.2115	12.61	0.0093	1	Yes
*B*-SF	1.04	1	1.0400	62.27	<0.0001	1	Yes
*C*-PPF	0.0464	1	0.0464	2.76	0.1403	1	No
*AB*	0.11	1	0.11	6.56	0.0375	1	Yes
*AC*	2.89 × 10^−6^	1	2.89 × 10^−6^	0.0002	0.9899	1	No
*BC*	0.2067	1	0.2067	12.32	0.0099	1	Yes
*A* ^2^	0.9628	1	0.9628	57.41	0.0001	1.01	Yes
*B* ^2^	2.25	1	2.25	133.93	<0.0001	1.01	Yes
*C* ^2^	1.09	1	1.09	65.26	<0.0001	1.01	Yes
Residual	0.1174	7	0.0168	—	—	—	—
Lack of fit	0.083	3	0.0277	3.22	0.1443	—	No
Pure Error	0.0344	4	0.0086	—	—	—	—
Cor Total	6.52	16	—	—	—	—	—

**Table 12:** ANOVA results for *Y*_3_.

**Source**	**Sum of Squares**	**df**	**Mean Square**	**F-Value**	***p*-Value**	**VIF**	**Significance**
*Y*_3_	0.4051	9	0.045	32	<0.0001	—	Yes
*A*-NS	0.1036	1	0.1036	73.64	<0.0001	1	Yes
*B*-SF	0.0458	1	0.0458	32.57	0.0007	1	Yes
*C*-PPF	0.0232	1	0.0232	16.47	0.0048	1	Yes
*AB*	0.0001	1	0.0001	0.0859	0.778	1	No
*AC*	0.0219	1	0.0219	15.59	0.0055	1	Yes
*BC*	0.0001	1	0.0001	0.1036	0.7569	1	No
*A* ^2^	0.1545	1	0.1545	109.82	<0.0001	1.01	Yes
*B* ^2^	0.0307	1	0.0307	21.8	0.0023	1.01	Yes
*C* ^2^	0.01	1	0.01	7.13	0.032	1.01	Yes
**Source**	**Sum of Squares**	**df**	**Mean Square**	**F-Value**	***p*-Value**	**VIF**	**Significance**
Residual	0.0098	7	0.0014	—	—	—	—
Lack of fit	0.0073	3	0.0024	3.75	0.1171	—	No
Pure Error	0.0026	4	0.0006	—	—	—	—
Cor Total	0.4150	16	—	—	—	—	—

**Table 13:** Model credibility testing.

Model	*R* ^2^	$R_{Adj}^{2}$	$R_{Pre}^{2}$	C.V./%	Adeq Precision
*Y*_1_	0.962	0.915	0.581	0.81	18.25
*Y*_2_	0.982	0.959	0.788	2.57	17.65
*Y*_3_	0.976	0.946	0.710	2.03	16.77

**Table 14:** Parameter value selection of MOPSO algorithm.

Parameters	Maximum Number of Iterations	Population Size	Pareto Solution Set Size	*w*	Inertial Weight Decay Rate	*C*_1_	*C*_2_	Mutation Selection Rate
Value	100	500	100	0.422	0.99	1.9775	1.7355	0.1

**Table 15:** The entropy values and weights of each evaluation index.

Evaluation Indicator	Entropy Value	Weight
*Y*_1_	0.98885	0.11636
*Y*_2_	0.93785	0.64835
*Y*_3_	0.97745	0.23529

**Table 16:** Multi-objective optimization results and verification.

Evaluation Indicator	Predicted Value	Experimental Value	Relative Error (%)
*Y*_1_/MPa	69.94	68.95	1.42
*Y*_2_/MPa	5.49	5.62	2.37
*Y*_3_	1.99	1.91	4.02

## Data Availability

The original contributions presented in this study are included in the article. Further inquiries can be directed to the corresponding author.

## Abbreviations

The following abbreviations are used in this manuscript:

NS	Nano-SiO_2_
SF	Steel fiber
PPF	Polypropylene fiber
NSDHFRC	Nano-SiO_2_-double-doped fiber high-strength concrete
RSM	Response surface model
MOPSO	Multi-Objective Probability-Based Simultaneous Optimization
BBD	Box–Behnken Design
CCD	Central Composite Design
SEM	Scanning electron microscopy
ANOVA	Analysis of variance
C.V.	Coefficient of variation
ITZ	Interfacial transition zone
